# Wellbeing Outcomes and Risk and Protective Factors for Parents with Migrant and Refugee Backgrounds from the Middle East in the First 1000 Days: A Systematic Review

**DOI:** 10.1007/s10903-023-01510-4

**Published:** 2023-07-06

**Authors:** Amelia Kate Winter, Clemence Due, Anna Ziersch

**Affiliations:** 1 School of Psychology, The University of Adelaide North Terrace, Adelaide, 5005 Australia; 2 College of Medicine and Public Health, Flinders University, GPO Box 2100, Adelaide, 5001 Australia

**Keywords:** Refugees, Migrants, First 1000 days, Maternal health, Wellbeing, Systematic review

## Abstract

The First 1000 Days (the period from conception to a child’s second birthday) is an important developmental period. However, little is known about experiences of parents with refugee and migrant backgrounds during this period. A systematic review was conducted according to PRISMA guidelines. Publications were identified through searches of the Embase, PsycINFO, PubMed, and Scopus databases, critically appraised, and synthesised using thematic analysis. A total of 35 papers met inclusion criteria. Depressive symptomatology was consistently higher than global averages, however maternal depression conceptualisations differed across studies. Several papers reported changes in relationship dynamics as a result of having a baby post-migration. Consistent relationships were found between social and health support and wellbeing. Conceptualisations of wellbeing may differ among migrant families. Limited understanding of health services and relationships with health providers may impede help-seeking. Several research gaps were identified, particularly in relation to the wellbeing of fathers, and of parents of children over 12 months old.

**Supplementary Information:**

The online version contains supplementary material available at 10.1007/s10903-023-01510-4.

## Introduction

The First 1000 Days is the period of time between conception and a child’s second birthday [1]. This period is known as an important developmental period for both parents/caregivers (henceforth ‘parents’) and infants, with consequences across the lifespan for both [2]. While there is a well-established body of literature concerning wellbeing outcomes during this time period for children [1, 3], to date, outcomes for parents in the First 1000 Days specifically have been underexplored, with literature focusing on the perinatal period (that is, pregnancy to the first six weeks post-birth) [4]. Moreover, most of the existing research has focused on people without migrant or refugee backgrounds [1]; therefore, the existing body of literature does not consider the specific needs parents with a refugee background may have during the First 1000 Days of their child’s development. As such, this systematic review aimed to explore: (1) the health and subjective wellbeing outcomes of parents with migrant or refugee backgrounds in the First 1000 Days of their child’s development, and (2) the risk and protective factors related to these wellbeing outcomes. The paper has a specific focus on people from the Greater Middle East given the extensive recent migration of people from this region.

### Definitions

It is important to note the difference between the terms ‘migrant’, ‘asylum seeker’, and ‘refugee’. Use of the term ‘migrant’ is most commonly in reference to a person who has permanently resettled in a separate country to their country of origin and made a free decision to migrate. ‘Asylum seeker’ indicates a person who is awaiting a decision regarding their application for refugee status, while ‘refugee’ refers to a person who meets the criteria for refugee status defined by the United National High Commissioner for Refugees (UNHCR) or that of a resettlement country [5]. While these definitions are important to note, unfortunately previous literature has not always clearly defined migration status. While every effort has been made in this paper to do so, this was not always possible. Therefore, the term migrant in reference to previous literature in this paper may include people with refugee and asylum seeker backgrounds.

This paper takes the World Health Organisation (WHO) definition of health as a complete state of mental, physical, and social wellbeing [6]. Additionally, The WHO defines psychological wellbeing (used interchangeably with the term ‘mental health’) as a state of wellbeing in which a person can cope with stressors, realise one’s own potential, and contribute to one’s community [7]. As such, this paper uses the term “wellbeing” to cover both the WHO definitions of health and psychological wellbeing.

### Background

Literature focusing on the general population has identified that the perinatal period (inconsistently defined in the literature but often pregnancy and the first year post-birth) is a vulnerable time for mothers who have been pregnant, both physically and mentally. In terms of physical health, changes to a woman’s body due to pregnancy can carry increased medical risk including a diverse range of issues such as gestational diabetes and hypertensive disorders [8]. In addition, many mothers are at increased risk for mental illness [9], with 13% of women worldwide experiencing a psychological disorder such as depression or anxiety in the perinatal period [10]. The World Health Organisation has specifically highlighted the importance of improving maternal mental health, pointing to a clear link between adverse mental health and maternal mortality and morbidity [11].

A small body of research has also explored more general wellbeing outcomes across the perinatal period. For example, a significant relationship has been found between feeling in control during labour and emotional wellbeing after birth [12], and exhaustion from lack of sleep has been linked to higher stress levels [13]. However, to date more general influences on wellbeing have been underexplored, with the majority of the research being conducted on clinically diagnosable conditions such as postnatal depression (PND) [14].

While men’s wellbeing specifically has not been studied at all in relation to the First 1000 Days, evidence related to men’s wellbeing outcomes in the perinatal period suggests men are also susceptible to negative mental health outcomes during this time [15], with men’s postnatal depression rates globally estimated at approximately 8% [16]. The transition to fatherhood can be stressful, due to changes in lifestyle, relationships with partners, shifting identity, and occupational stress [17–19].

In terms of literature concerning migrant and refugee women, physical maternal health outcomes have been shown to be less favourable than general populations, owing to issues such as barriers to accessing healthcare, and increased likelihood of complex presentations and comorbidities [20, 21]. In relation to mental health, a systematic review of the literature on PND rates in migrant women [22] showed that rates of PND were as high as 42%, much higher than the global average of 13% [11]. Similarly, a 2008 Canadian study found that migrant, asylum seeker, and refugee woman were approximately three times more likely to screen positive on the Edinburgh Postnatal Depression Scale (EPDS) [23], a common screening tool for depressive symptomology used worldwide. There is little literature for fathers, however an Australian study of fathers of young children found that fathers with a refugee or non-English-speaking migrant background in general were more likely to experience psychological distress and to report worse physical health [19].

However, much of the literature on migrant and refugee wellbeing outcomes in the First 1000 Days does not delineate between regions of origin, nor does it focus on a broader holistic understanding of wellbeing. As such, wellbeing outcomes for parents during the first 1000 days who have migrated from the Greater Middle East as long-term migrants are not clear. This is an important gap because many people from the Greater Middle East have been exposed to significant pre-migration stressors, resulting in high levels of trauma, changes in family dynamics, and sexual violence particularly for women [24]. In turn, such stressors have been associated with negative wellbeing and reproductive health outcomes [22, 25]. Whilst there is variation between groups within the Greater Middle East, a focus on this region is important to understand the overlapping cultural factors specific to this region that may influence wellbeing in the First 1000 Days.

## Aims

Taking the broad view of wellbeing defined above, this paper addresses two research questions: (1) what are the wellbeing outcomes of migrant and refugee parents from the Greater Middle East in the First 1000 Days of their child’s development?; and (2) what are the risk and protective factors associated with these wellbeing outcomes?

## Methods

This systematic review was conducted in accordance with the Preferred Reporting Items for Systematic Reviews and Meta-Analyses (PRISMA) guidelines [26]. A detailed protocol was developed and registered on the international prospective register of systematic reviews (PROSPERO) database: registration number CRD42020187153.

### Eligibility Criteria

All peer-reviewed, empirical studies published in English that reported on any wellbeing outcome that fitted the broad definition provided above, and/or associated risk and protective factors, of migrant or refugee parents from the Greater Middle East geopolitical region, consisting of Afghanistan, Algeria, Armenia, Azerbaijan, Bahrain, Comoros Islands, Cyprus, Djibouti, Egypt, Eritrea, Georgia, Iran, Iraq, Israel, Jordan, Kazakhstan, Kuwait, Kyrgyzstan, Lebanon, Libya, Mauritania, Morocco, Oman, Palestine (West Bank and Gaza strip), Pakistan, Qatar, Saudi Arabia, Somalia, Sudan, Syria, Tajikistan, Tunisia, Turkey, Turkmenistan, United Arab Emirates, Uzbekistan, Western Sahara, and Yemen [27] in the First 1000 Days were eligible for inclusion. Outcomes of interest were any wellbeing (including physical, psychological, or social) impact occurring as a result of having a child in the past two years, while risk and protective factors were defined as factors that contribute to vulnerability or enhance wellbeing, respectively [28]. No restrictions were placed on publication dates. Studies focusing on the outcomes of infants were excluded as this paper aimed to explore wellbeing outcomes for parents only. Given the differences in reporting refugee status across the literature and the tendency for much of the literature to not distinguish between migrant and refugee participants, all studies of parents migrating internationally from the Greater Middle East were eligible for inclusion. While a limitation of this approach is the inability to compare outcomes according to migration status, this study maintained a broad approach to inclusion to avoid excluding relevant studies. Studies focusing on internal migration or where migration status was unclear were also excluded; participants were required to have been born in the Greater Middle East and to have had a pregnancy or baby post-resettlement for papers to be included. Where papers met all other criteria, but it was unclear whether participants were in transit or had been resettled, the study was included in the review. Likewise, where papers included data from various regions but the Greater Middle East-specific data could be extracted, these were included; papers where Greater Middle East-specific data could not be extracted were excluded e.g. [29]. Systematic or scoping reviews, theses, conference papers, and case studies were all excluded.

### Search Strategy and Data Extraction

A search strategy was developed and employed with the assistance of a university research librarian. Four electronic databases were searched: Embase, PsycINFO, PubMed, and Scopus. Search terms pertained to pregnancy, postpartum, and First 1000 Days; refugee and migrant status; and physical and psychological wellbeing. While this review focuses specifically on the Greater Middle East, no geographical terms were included in the search to ensure broad reach, with this occurring in the screening process. See Supplement 1 for search strategies for each database. The final search was conducted on 28th July 2022.

The search identified 3,774 articles after removing duplicates. Titles and abstracts were screened by the first author using Covidence [30], a screening and data extraction tool. Full text screening of 868 papers was then conducted together with the second and third authors, with an inter-rater agreement of 80%; discrepancies were resolved through group discussions between all authors, by comparing papers together against inclusion criteria. This resulted in a final sample of 35 papers. Data was extracted according to a predesigned table (see Supplement 2).

No meta-analysis was performed due to the heterogeneity of study designs amongst the included papers. Instead, findings from the included papers were synthesised according to the principles of thematic analysis [31, 32] by the first author, in collaboration with all authors; all of whom are experienced in this type of analysis. Specifically, an inductive and deductive approach was taken, whereby all articles were read and findings regarding wellbeing outcomes and/or risk and protective factors for parents were coded by the first author and then collated into overarching themes. Any disagreements regarding codes and themes were resolved through meetings between all authors to achieve unanimous agreement.

**Fig. 1:**
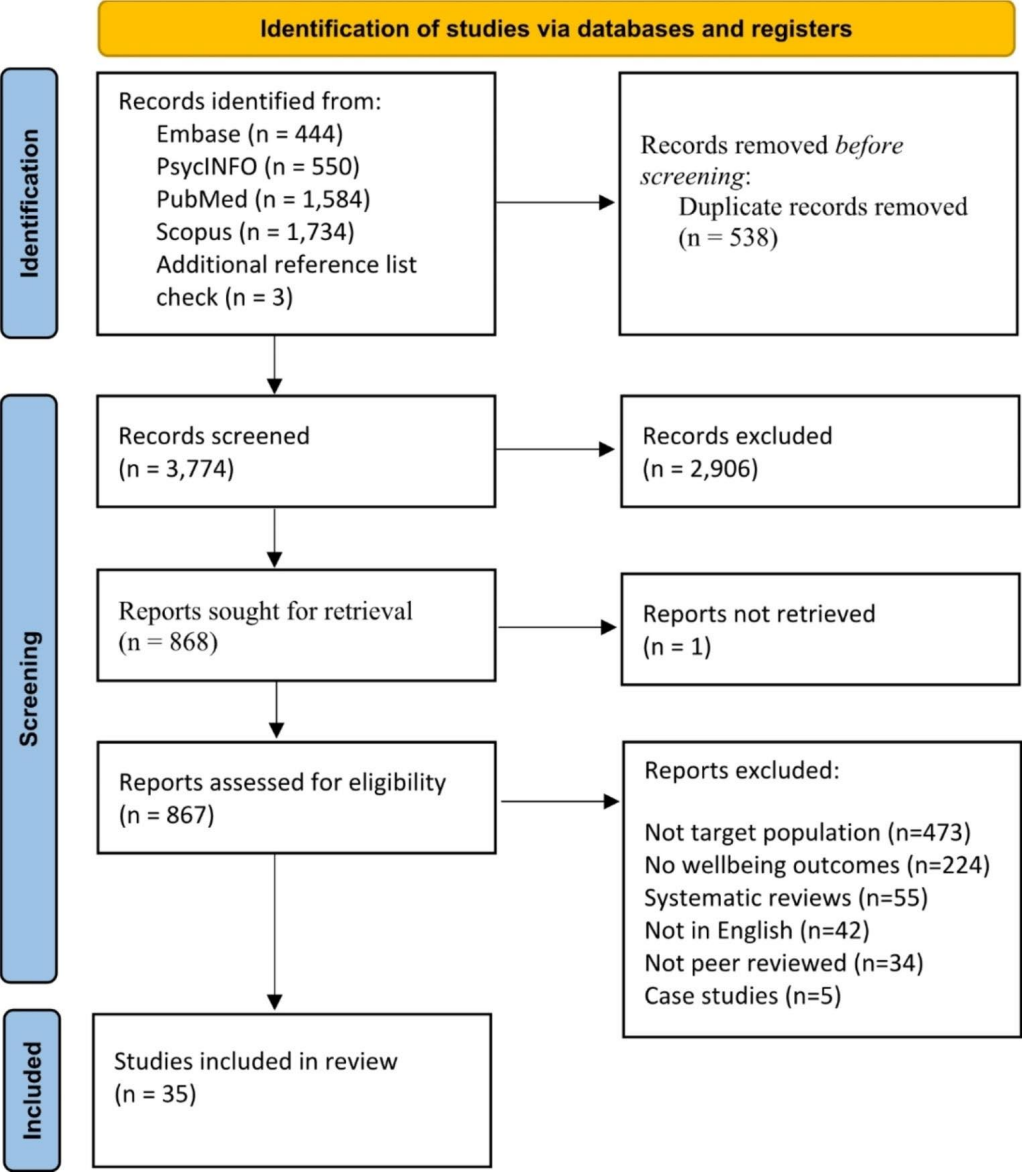
Search flowchart

### Quality and Bias

Study quality was assessed using the Mixed Methods Appraisal Tool (MMAT) [33]. The first author assessed the quality of the papers, and the second and third authors reviewed this to confirm findings. No article was excluded on the basis of quality given the small number of studies which met the inclusion criteria.

## Results

### Description of Included Papers

From the initial 3,774 papers identified in the search, 35 met the inclusion criteria (see Supplement 2 for a summary of all included papers). Papers were published between 1999 and 2022. Table 1 provides the characteristics of the included papers. Fourteen papers were quantitative [34–47]; 19 were qualitative [48–66]; and two were mixed methods [67, 68]. One paper was qualitative but included a measure of depression for context [66]. Sixteen papers specified participants were refugees [37–39, 41, 43, 46, 54, 58–61, 63–67]; the remaining papers included participants of both refugee and migrant status, or did not specify. Because more than half of the included papers did not specify refugee status, it was not possible to consistently examine differences between refugees and other migrants. 30 papers were conducted in resettlement countries [34–37, 39, 40, 42, 44–46, 48–53, 55–66]; three papers were unclear as to whether participants were in transit or had permanently resettled [38, 41, 54]; and two studies included participants who had both registered for resettlement (though permanency was unclear), and who had resettled without documentation [43, 47]. In five instances two or more papers included the same data and thus appeared to be from the same overarching study: two papers by Alhasanat-Khalil and colleagues [35, 36], two papers by Bawadi and colleagues [48, 62], three papers by Hjelm and colleagues [51–53], two by Riggs and colleagues [58, 59], and two by Stirling Cameron and colleagues [64, 65]. Therefore, while 35 papers are included in this review, the data represents 29 individual studies. In synthesising the data, these papers have been treated as separate; however, where necessary this issue has been noted.

Only one paper [57] specifically explored the perspectives of men. Three papers [58, 59, 61] explored the perspectives of both men and women.

Resettlement occurred in a range of countries. Twenty-six papers presented data collected in high income countries, namely Canada [40, 42, 44, 45, 64, 65, 67], USA [34–36], United Kingdom [48, 62], Sweden [49, 51–53, 56, 57], Australia [50, 55, 58–61], and Germany [63]. The remaining papers were conducted in middle-income countries, namely Jordan [37, 41, 46], Turkey [39, 54, 66], Lebanon [38, 43], and Iran [47]. Seven papers did not specify country of origin of all participants other than ‘Middle East’ or ‘Maghreb’ [35, 36, 40, 42, 44, 45, 57]. All other papers specified the countries of origin of participants; these countries were Syria, Yemen, Lebanon, Iraq, Egypt, Jordan, Qatar, Saudi Arabia, Algeria, Sudan, Morocco, Afghanistan, Palestine, and Turkey.

**Table 1:** Overview of included studies

	Quantitative (*N* = 14)	Qualitative (*N* = 19)	Mixed methods (*N* = 2)	Total (*N* = 35)
**Migrant status**				
Refugee	7	8	1	16
Migrant/not specified	3	11	1	15
Both refugee and migrant (delineated)	4	-	-	4
**Outcome category**				
Mental	8	14	2	24
Physical	4	3	-	7
Both mental and physical	2	2	-	4
**Sample size of ME participants**				
< 10	-	6	-	6
11–50	1	13	2	16
51–100	3	-	-	3
101–200	2	-	-	2
201–300	-	-	-	-
301–500	3	-	-	3
500+	5	-	-	5
**Participants**				
Women	14	15	2	31
Men	-	1	-	1
Health providers, women, and men	-	3	-	3
**Country of resettlement**				
Australia	-	6	1	7
Canada	4	2	1	7
Germany	-	1	-	1
Iran	1	-	-	1
Jordan	3	-	-	3
Lebanon	2	-	-	2
Sweden	-	6	-	6
Turkey	1	2	-	3
United Kingdom	-	2	-	2
United States of America	3	-	-	3

### Quality of Included Papers

Critical appraisal of the included papers was performed using the Mixed Methods Appraisal Tool (MMAT) [33], which considers clarity of research questions, appropriateness of data, and appropriateness of sampling, measurement, and analysis methodology [33]. Included papers were assessed in their published form to consider the quality of the published literature; authors were not contacted for further information. Therefore, it is important to note that reporting issues may be attributed to word counts or other publishing requirements.

All papers had clear research questions, and all studies included data that addressed the research questions, thereby satisfying the first MMAT screening criteria. All qualitative papers used appropriate data collection and analysis techniques. However, only three papers [49, 61, 67] included the interview schedule. Six papers [48, 51, 58, 60, 64, 65] included an exemplar of the interview schedule. The remaining nine qualitative papers provided no details of the interview questions.

All quantitative papers reported relevant data that answered the research questions, and all papers articulated a relevant sampling strategy. The two mixed methods papers [67, 68] provided adequate rationale for employing mixed methodology and sufficiently integrated data.

In terms of sampling five were cohort studies [37, 39, 40, 44, 45]; all other papers employed convenience or purposive sampling methods, such as recruitment through health services, which may be a potential source of bias.

Of the studies which reported funding, none reported a conflict of interest, with funding predominantly from government bodies, philanthropic organisations or universities.

### Findings in Relation to Emotional Wellbeing, Including Risk and Protective Factors

#### Depression, Anxiety, and Emotional Distress

In studies where ante- and post-natal depressive symptomology was measured (11 in total), prevalence rates ranged from 22 to 43% antenatally and 25–57% postnatally. Four of these papers focused specifically on Syrian women [41, 43, 66, 67]; one focused specifically on Afghan women [68]; one paper reported on women from Egypt, Jordan, Lebanon, Palestine, Qatar, Saudi Arabia and Yemen [34]; and four papers did not specify country of origin other than ‘Middle East’ [35, 36, 40, 42]. It should be noted that the four papers on Syrian women [41, 43, 66, 67] specifically described these women as refugees, while the other papers did not describe migration context, or did not distinguish between refugee and migrant status within the data. Higher prevalence was observed in papers reporting specifically on Syrian refugee women than in other studies included in this review (see Table 2 for further details), however as noted given the heterogeneity of research methods, no statistical analysis was completed. One paper [43] did not differentiate between ante- and post-natal participants for an overall prevalence of 74.3% in Syrian refugee women.

**Table 2:** Prevalence of antenatal and postnatal depression

Study	Measure	Prev.	Population
**Antenatal depression**			
Ahmed et al. [67] Miszkurka et al. [40] Peer et al. [42]	EPDS ≤ 10 CES-D ≤ 16 EPDS ≤ 12	43% 22% 24%	Syrian refugee women ‘Middle East’ not further specified ‘Middle East’ not further specified
**Postnatal depression**			
Ahmed et al. [67] Alhasanat et al. [34] Alhasanat-Khalil et al. [35] Alhasanat-Khalil et al. [36] Mohammad et al. [41] Qutranji et al. [66] Shafiei et al. [68]	EPDS ≤ 10 EPDS ≤ 10 EPDS ≤ 10 EPDS ≤ 10 EPDS ≤ 12 PHQ-9 ≤ 5 EPDS ≤ 13	57% 36% 25.2% 25.2% 49.6% 44.8% 31%	Syrian refugee women Egypt, Jordan, Lebanon, Palestine, Qatar, Saudi Arabia, Yemen ‘Middle East’ not further specified ‘Middle East’ not further specified Syrian refugee women Syrian refugee women Afghan women
**Reported ante/postnatal combined**			
Stevenson et al. [43]	EPDS ≤ 13	74.3%	Syrian refugee women

Nine papers explored emotional distress or depressive symptomatology qualitatively [50, 54, 55, 61, 64–68]. While some papers found that Greater Middle Eastern women often discussed outcomes consistent with depression (e.g., sadness, sleep disturbance, social withdrawal) [50, 55], these experiences were not always considered ‘depression’ by participants – as in the case of Syrian women in Ahmed et al.’s research who instead conceptualised such emotions as boredom [67]. Importantly, distress postnatally was also related to migration by participants in some papers – for example, one paper [54] found that Syrian refugee women living in Turkey discussed distress in terms of fear of birth, fear of dying, and nightmares as a result of fleeing a war.

Seven articles mentioned unplanned or unwanted pregnancies [34–36, 38, 41, 46, 54], with two exploring the relationship with wellbeing. The Khawaja and Hammoury[38] paper on sexual coercion of Palestinian women living in Lebanon found that 31.9% of the 349 participants did not desire their current pregnancy and this was significantly associated with forced sexual intercourse. Of the 365 Syrian women living in Jordan who participated in the Mohammad et al. paper [41], 251 women (68.8%) did not plan to become pregnant. However, this was not significantly related to an EPDS score of 12 or above.

One qualitative paper [49] reported women’s experiences following a foetal diagnosis, such as trisomy 21, finding that women described feelings of sadness, loneliness, sorrow, shock, and grief.

#### Loneliness, Isolation and Social Support

Ten papers discussed women’s [50, 54–56, 60, 64–66, 68] and men’s [57] experiences with feelings of loneliness and isolation postnatally. One paper was mixed methods [68]; all others were qualitative. Four of these papers used data from the same study: two papers by Hjelm and colleagues [51, 52] and two by Stirling-Cameron and colleagues [64, 65]. Each of these studies found that women frequently reported experiencing isolation, loneliness, and homesickness; these factors were specifically reported as primary contributors to sadness or depression by participants in three papers [50, 66, 68]. One study [64] explored birth and postnatal experiences of Syrian refugee women in Canada during the COVID-19 pandemic; stay at home orders in particular exacerbated experiences of loneliness and isolation. While the one paper which considered men in relation to loneliness [57] found that men did not explicitly discuss loneliness and isolation after having a baby, participants did discuss isolation in relation to unemployment and feelings of weakness, lack of productivity, and lack of respect following the birth of a baby in a resettlement country.

Relatedly, 18 papers discussed social support as either a risk or protective factor for physical or emotional wellbeing for Greater Middle Eastern parents. Three papers were quantitative [34, 35, 41], 13 papers were qualitative [50–52, 54–56, 59–61, 63–66], and two papers were mixed methods [67, 68]. The qualitative papers found that women reported emotional, religious and practical support after birth to be protective, but some participants noted that they were more supported in their home countries [50]. In terms of the quantitative findings, lack of social support was identified amongst five significant risk factors for an EPDS score above ten [34], while acculturative stress and lack of social support were found to be predictors of postnatal depressive symptomatology, particularly for women who experienced marginalisation [35]. A significant correlation was also found between low levels of social support and higher EPDS scores [41].

### Findings in Relation to the role of Parent, and Family Roles and Dynamics

#### Family Dynamics

Five qualitative papers[48, 50, 54–56] discussed meanings of being a mother, and the role of mother in relation to wellbeing. In general, they indicated that women were considered by participants to be ‘natural’ mothers within their cultural background [50, 55, 56], although this was not always considered an easy role. Participants in two papers [50, 54] indicated that becoming a mother in a resettlement country (in this case Australia and Turkey) gave them a new purpose post-resettlement.

Five qualitative papers [48, 56–58, 60] described changes in relationship dynamics as a result of having a baby in a resettlement country. Such changes included a shift in focus from extended to nuclear family, changes in roles of mother and father, and changes in the marital relationship.

Five qualitative papers [48, 56–58, 60] found that there was an increased involvement of fathers within the family in resettlement countries compared to countries of origin. There were mixed findings in these papers; however, attendance at childbirth - while not always seen as the norm culturally - was seen as an improvement in practice after resettlement for both men and women that positively affected wellbeing [56, 57, 60]. Increasing men’s involvement in childcare more generally, however, was not always seen as positive by the participants in these papers. For example, six of the seven Iraqi women in the Di Ciano et al.[50] paper maintained that childrearing was the mother’s responsibility, and they did not wish to pressure their husbands to take on further responsibilities. However, some women in this paper also reported a sense of helplessness and lack of support because traditional gender roles within families dictated that husbands were not available to provide practical support within the household [50]. Additionally, in a qualitative investigation of Middle Eastern men’s experiences of maternal health care in Sweden, some men reported an increased dependence on them from their wives, and others said they felt weak and burdensome to their wives as a result of unemployment [57].

Six papers included discussion around intimate partner violence (IPV); five quantitative [38, 43, 46, 47, 67] and one qualitative [61]. Ahmed et al. [67] used the short form Women Abuse Screening Tool (WAST) [69] as part of their larger study on prevalence of maternal depression in Syrian refugee women living in Canada. No participants responded in the affirmative; these participants also reported receiving social support from their partners. Khawaja and Hammoury [38], in their quantitative paper on sexual coercion within marriage among pregnant Palestinian refugee women in Lebanon, employed the Abuse Assessment Screen (AAS) [70], and found that of 349 pregnant women, 19.1% of women reported being physically abused by their husband in the last year and 26.2% of women reported being forced to have sexual intercourse within the past year. The Stevenson et al.[43] study assessed IPV via the answer to a single question, “have you experienced domestic violence?”. The study found that women from Syria residing in Lebanon who had been exposed to IPV had significantly higher scores on the EPDS. The Nabolsi et al.[46] paper reported violence experience as a yes/no question; no further details were reported. 6.5% of women reported violence; lack of violence experience was a significant predictor of improved outcomes on the HRQoL general health subscale. The Dadras et al. [47] paper on Afghan refugee women in Iran assessed intimate partner and/or family violence with the single question, “have you ever been physically, sexually, emotionally, or verbally abused by your partner or someone close to you during your pregnancy?”; 15.5% of women reported violence and these women were more likely to experience pregnancy complications. Finally, Yelland et al.[61] asked about violence at home as part of their interview schedule with health care providers (HCPs). HCPs reported that approaching the issue of violence was the most difficult part of a social health assessment. They did not explore prevalence.

One paper described the exacerbation of a mother’s loneliness and isolation due to the social control imposed by her husband [66].

### Obstetric Outcomes

Eight papers reported on the physical health outcomes of mothers during pregnancy and the postnatal period. Of these papers, three from the same overarching study [51–53] concerned gestational diabetes, three papers [37, 39, 47] assessed delivery and obstetric outcomes, one paper [44] assessed preeclampsia rates, and one paper[50] reported on general physical health of mothers postnatally.

One quantitative paper from Jordan [37] compared birth outcomes of a cohort of 616 Syrian refugee women with 644 Jordanian women and found that 58.9% of Syrian women were anaemic compared with 33.4% of Jordanian women, and that Syrian women were more likely to give birth by caesarean section than Jordanian women. Syrian women also had a significantly lower time between hospital admission and delivery; however, it was unclear whether this was due to delays in seeking care, or true precipitate labour [37]. Another quantitative paper from the same study [39] found that, compared to 940 Turkish women, the 616 Syrian refugee women participants were less likely to experience pre-eclampsia (1.62% versus 5.32% for Turkish women); Hemolysis, elevated liver enzymes, and low platelet count (HELLP Syndrome) (no Syrian women experienced HELLP, versus 1.28% for Turkish women); or placental anomalies (no Syrian women experienced placental anomalies, versus 1.28% for Turkish women), and there were no significant differences in haemoglobin levels or cholestasis between cohorts. Similarly, a quantitative paper [44] evaluated migrant women’s risk of pre-eclampsia in Canada and found that the 8,552 women from the Middle East and North Africa included in the study were not more likely than migrants from other nations to experience serious preeclampsia (OR = 1.03, CI = 0.58–1.84 for women with prior live births; and OR = 1.16, CI = 0.59–2.32 for women without a prior live birth). The paper on Afghan women in Iran [47] found that 56.6% of women in the study experienced at least one pregnancy complication (including pre-term labor, pregnancy loss, stillbirth, eclampsia/pre-eclampsia, hypertension, gestational diabetes, intrapartum haemorrhage, or infection). Higher parity, more recent migration, undocumented migration status, lack of health insurance, lower income, IPV, and poor mental health were all associated with increased occurrence of adverse pregnancy outcomes [47].

In the qualitative paper on Iraqi women living in Australia [50], women reported physical difficulty after birth, particularly during recovery from caesarean section. Women said this was exacerbated by a lack of practical support during recovery. The qualitative papers [51–53] of Swedish and Middle Eastern (Iraqi, Iranian, and Lebanese) women with a gestational diabetes mellitus (GDM) diagnosis found that women from the Middle East were more liked than Swedish women to express anxiety related to GDM and their baby’s health outcomes, but less likely to have knowledge concerning GDM including the causes[51] and the possibility of developing Type 2 Diabetes later in life[52].

### Experiences of and Barriers to Healthcare Provision

In terms of experiences of healthcare, ten papers – all qualitative - discussed the overall experiences of parents engaging with maternity and early childhood services[49, 50, 54, 56, 58–60, 63, 68]. Di Ciano et al.[50], Russo et al.[60], Riggs et al.[59] and Korukcu et al.[54] all found that support from HCPs was viewed favourably by women during and after pregnancy, and the majority of participants were satisfied with the care they had received. In particular, the standard of medical care was often compared favourably to that of women’s countries of origin. Findings by Ny et al. [56], Carlsson et al. [49], and Henry et al.[63] were mixed. These papers focused on Middle Eastern women living in Sweden [49, 56] and Germany [63], and found that while some participants felt the perinatal care received in Sweden or Germany was more comprehensive, other women felt dismissed and unsatisfied. Likewise, some participants in the Shafiei et al. [68] paper felt maternity HCPs in Australia were rushed. Men’s experiences of healthcare provision was only explored in two Australian papers on Afghan men [58, 61]. Both found that participants felt their own concerns were not addressed by maternity HCPs, as the focus was on their wives.

Four papers discussed lack of awareness of available services as a clear barrier to physical and mental health service provision postnatally [50, 55, 68]. One study [55] noted that while women were grateful to receive information about postnatal depression because it facilitated help-seeking, they felt the existing education and support were not sufficient. Likewise, Afghan women interviewed in another qualitative paper [68] reported their experiences being minimised by HCPs when they mentioned emotional wellbeing concerns after having their baby. Notably, one woman specified HCPs’ negative attitudes towards Muslim women as a barrier to help-seeking [68]. Likewise, several women in another paper [62] relayed experiences of discrimination and prejudice that interfered with both their care, and the care of their infant.

Stigma was described as a barrier to mental health help-seeing antenatally and postnatally in two papers [55, 67]. This included stigma from women’s communities and families, and made women concerned about accessing care, particularly through interpreters where privacy and confidentiality might be breached. Additionally, one participant in the Stirling Cameron [65] paper relayed being denied a referral to a mental health care professional by her family physician.

Language barriers to seeking or gaining appropriate care both before and after a baby was born were discussed in thirteen papers[49, 50, 53, 54, 56, 58, 59, 61–66]. This included delays in getting appropriate information[50, 63], difficulties making healthcare decisions [49, 63], unsatisfactory experiences with interpreters such as difficulties making appointments [53, 56, 64], interpreters not being available in the dialect that participants spoke [58], and requesting female interpreters but getting men, leading to discomfort[61]. Three papers [57, 58, 62] found these barriers led to husbands interpreting for their wives.

One paper specifically investigated COVID-19-related barriers [64]. Syrian women living in Canada reported the suspension in Canada of doula support and the lack of recognition of doulas as healthcare staff, limiting the available support and exacerbating existing barriers to healthcare [64]. Women in this paper also reported heightened anxiety caused by fear of COVID-19, and grief regarding unmet expectations of how their pregnancy and birth would be [64].

## Discussion

This is the first systematic review to consider the physical and emotional wellbeing outcomes for new parents from the Greater Middle East in the First 1000 Days of their child’s development in the context of resettlement. Overall, the review found that parents from the Middle East experience additional challenges when having a baby in resettlement countries, and this has the potential to exacerbate existing stressors - although sometimes changes to support and other structures had more positive impacts. Specifically, the review found that Greater Middle Eastern migrant women have a higher risk of postnatal depression than the general population, that new parents from the Greater Middle East are likely to experience loneliness and isolation in resettlement countries, that experiences and expectations of care during and after pregnancy may differ on cultural bases, that the experience of having a baby post-resettlement presents opportunities and challenges for family life, and that social support and understanding of the migration context are crucial to supporting migrant families from the Greater Middle East during the First 1000 Days.

The findings of this review indicate that, while PND rates varied considerably between papers, migrant women from the Greater Middle East may be at higher risk for developing PND which is consistent with previous literature for migrant women overall [23]. Three papers [41, 43, 67] indicated that refugee women from Syria may be at particularly high risk, which supports previous literature regarding the increased likelihood of mental illness in refugee populations more generally [71]. However, there may be issues related to the assessment of PND for Middle Eastern women, including heterogeneity in screening methods for PND. The EPDS, the postnatal depressive symptomology screening tool employed in the majority of papers measuring PND symptoms, is generally considered valid and reliable in women from the Greater Middle East [72–74]; however, comparing findings across papers in this review was difficult due to varying cut-off scores. Importantly, qualitative papers indicated Greater Middle Eastern women often conceptualise distress postnatally differently to the criteria included in the EPDS. Therefore, while women may not screen as high-risk on a screening instrument, they may still self-report depressive symptomatology, and this should not be ignored.

Separate from PND screening, the papers included reported a wide range of factors that impacted the wellbeing of mothers and fathers. Loneliness and isolation were the two most apparent. However, several papers in the review indicated the positive wellbeing effects migration can also bring about, particularly for new mothers, as their self-sufficiency may increase post-resettlement. Additionally, women reported positive wellbeing outcomes in the context of the thorough maternity care they received in resettlement countries. However, limited understandings of the available health services, as well as concerns about engagement and support from HCPs, were found to impede help-seeking, particularly for issues concerning emotional wellbeing [50, 55, 56, 65]. This is consistent with findings of a systematic review of migrant women’s maternity care experiences by Fair and colleagues [75] that called for new models of maternity care with increased cultural competence and greater consideration for migrant women’s wellbeing.

Studies that included fathers focused on wellbeing in relation to changing roles post-migration [57–59]. However, none of the included papers addressed PND for fathers. Like motherhood, the transition to fatherhood is known to be an important time for men, with its own potential effects on wellbeing [76]. Given the added layer of complexity regarding wellbeing for migrant families post-resettlement, an understanding of the wellbeing impacts on fathers is crucial. Similarly, emerging evidence demonstrates the impact pregnancy and infant loss has on men’s emotional wellbeing[77], however there is currently no data on this in a resettlement context for men or women.

Five papers[38, 46, 47, 61, 67] discussed intimate partner violence (IPV), and one discussed social control by husbands (specifically, preventing wives from accessing social supports) [66]. One study[38] specifically explored sexual coercion. Notably, there was a change across papers: more recent papers were more likely to include IPV discussion. Each paper conceptualised IPV in a different way, making it difficult to compare. Additionally, one paper [67] had no participants report IPV, in contrast to the other relevant papers; in this paper, participants positively described the social support they received from partners. It should be noted, however, that previous literature suggests IPV may be particularly under-reported in migrant populations, where economic and cultural barriers may prevent women from reporting IPV [78]. Additionally, general prevalence rates of IPV in this systematic review were in line with WHO estimates and previous research involving migrant families more broadly [79]. WHO estimates of intimate partner violence in the Eastern Mediterranean region (Egypt, Iran, Iraq, Jordan, and Palestine), are higher than the global average at 36.4% [80]. Violence during pregnancy has been found to be a risk factor for poorer physical and psychosocial outcomes for women and their infants [80]. As such, it is clear that more research is needed into IPV for migrant families from the Greater Middle East.

Interestingly, overall, obstetric outcomes for women from the Greater Middle East giving birth post-resettlement were not found to be problematic. Risk of serious complications such as preeclampsia, HELLP, or cholestasis, was not higher than the general population, and in some cases was lower [39]. This may support previous findings of the “healthy migrant effect”, whereby migrants demonstrate more favourable health outcomes post-resettlement as a result of self-selection: given the physical, emotional, and financial demands of migration, individuals with greater resources and therefore improved health may be more likely to migrate [81]. However, the Alnuaimi et al.[37] paper found that Syrian women were more likely to be anaemic than their Jordanian counterparts, and that the time between presenting at hospital and giving birth was significantly lower. This may be related to refugee status (all the Syrian women were refugees); however, future research is needed to ascertain whether this is an issue of broader health vulnerability or inadequate maternity care.

Six papers mentioned unplanned pregnancy in the results; however, no analysis was performed on the relationship between unplanned pregnancy and wellbeing in any of the papers. Previous research suggests a greater psychosocial risk profile for women whose pregnancies were unplanned [82] and thus this is a clear direction for future research. As with IPV, more recent papers were more likely to include discussion of unplanned pregnancy as a factor affecting wellbeing.

Overall, papers included in the review reported high satisfaction regarding the maternity and early childhood care received by participants. However, consistent with previous research on maternity care for migrant women[75], language barriers, lack of awareness of health services, and stigma related to mental health were all risk factors for wellbeing across this time period. Supporting previous research on the centrality of consent and control in the perinatal period to wellbeing [12], this review found that processes and procedures during perinatal care are not always adequately explained to migrant or refugee women. The results of this review also indicate that men are currently overlooked in maternity care.

One paper specifically investigated the impacts of COVID-19 [64]. As emerging research suggests several impacts of COVID-19 on perinatal and wellbeing outcomes, further research is needed.

All papers met the screening criteria for the MMAT. However, key areas of improvement with regard to quality were evident, particularly the inclusion of interview questions for qualitative and mixed methods papers, and ensuring all variables are clearly defined.

While this systematic review provides a thorough overview of the wellbeing outcomes and risk and protective factors specific to parents from the Greater Middle East post resettlement, it has several limitations. Firstly, only articles published in English were included in the search strategy. This may introduce a source of bias. Secondly, while this study sought to examine wellbeing outcomes and risk and protective factors for parents in the First 1000 Days of their child’s development, the results of this review must be considered in relation to the timeline of the actual papers. Very few papers actually included the full two years post birth, with the majority of papers reporting up to 12 months post-birth. Likewise, very few studies included early pregnancy. This highlights a gap in the literature around the wellbeing outcomes for parents during the First 1000 Days specifically, and is a direction for future research. Additionally, while the definition of the Greater Middle East was broad to capture more detail, people from this region are diverse and their attitudes, beliefs, and practices vary widely. Additionally, the papers in this review investigated outcomes in a variety of resettlement contexts each with their own specific health system and varying entitlements for migrants and refugees. Differences in the health care contexts of resettlement countries, for example between high income and middle income countries or public versus private health systems, health service entitlements for participants, and migration status of participants, are important to consider and limit the applicability of these findings to all contexts. A greater pool of studies would assist in teasing apart these limitations to understand wellbeing outcomes for refugee and migrant women from the Greater Middle East in the First 1000 Days of their child’s development, and thus more research in this area is needed. Likewise, the heterogeneity of study designs made it challenging to draw substantial conclusions regarding wellbeing outcomes, particularly physical outcomes, specific to migrant parents from the Greater Middle East. In addition, in five cases, data from one study had been split across papers, thereby reducing the sample for this review. This heterogeneity is further impacted by the differences in health contexts between resettlement countries of papers included in this review; the final papers including data from Australia, Canada, Germany, Sweden, United Kingdom, United States of America, Iran, Jordan, Lebanon, and Turkey. Finally, this review found inconsistencies in the literature regarding the categorisation of people with a migrant background, which may lead to the specific needs of refugee parents not being identified. Given the differences in experience between migrant and refugee populations – for example, choice of resettlement country, potential uncertainty regarding temporary versus permanent resettlement, and other pre- and post-resettlement stressors impacting physical and psychosocial wellbeing that may be associated with refugee status [83, 84] - further research considering wellbeing outcomes and related risk and protective factors for refugees specifically is recommended.

## Conclusion

The First 1000 Days presents a range of challenges for new parents. Families who have migrated from the Greater Middle East face additional challenges post-resettlement when welcoming a new baby, including changes to family dynamics, less social support than in home countries, and a lack of understanding of health services, all of which have the potential to negatively affect wellbeing. The provision of supportive, client-centred health care and comprehensive physical and mental health information is essential to support parents’ wellbeing, as is a well-rounded understanding of the individual contexts, health beliefs, and cultural belief and practices of families engaging with maternity and early childhood health services.

### Electronic supplementary material

Below is the link to the electronic supplementary material.

**Supplement A**. Search strategy for each database.

Supplementary Material 2
