# Supplementary material for: Wellbeing Outcomes and Risk and Protective Factors for Parents with Migrant and Refugee Backgrounds from the Middle East in the First 1000 Days: A Systematic Review
Source: J Immigr Minor Health. 2023 Jul 6;26(2):395–408. doi: 10.1007/s10903-023-01510-4 (PMC10937786; doi:10.1007/s10903-023-01510-4)
Supplement: Supplementary file 1 — Supplement A. Search strategy for each database. [file 10903_2023_1510_MOESM1_ESM.docx]

Supplement A

*Search strategy for each database*

| Database | Search terms |
| --- | --- |
| Embase  PsycINFO  PubMed  Scopus | migrant/exp OR “ethnic group”/exp OR “culturally and linguistically diverse”:ti,ab OR resettlement:ti,ab OR “displaced person*”:ti,ab AND “perinatal period”/exp OR pregnancy/exp OR infant/exp OR toddler/exp OR “preschool child”/exp OR “postnatal development”/exp OR “first thousand days”:ti,ab OR “first 1000 days”:ti,ab OR “early life”:ti,ab AND “mental health”/exp OR stress/exp OR “psychosocial environment”/exp OR wellbeing/exp OR “anxiety disorder”/exp OR emotion/exp OR “perinatal depression”/exp AND Parent/exp OR parenting:ti,ab OR “child parent relation”/exp OR mother/exp OR father/exp OR matern*:ti,ab OR patern*:ti,ab  Exp refugees OR refugee*.ti,ab OR migrant*.ti,ab OR immigrant*ti,ab OR asylum seeker*.ti,ab OR culturally #nd linguistically diverse.ti,ab AND Exp perinatal period OR exp postnatal period OR antenatal.ti,ab OR child*.ti,ab OR infant*ti,ab OR birth OR pregnan*ti,ab OR first thousand days.ti,ab OR first 1000 days.ti,ab AND Wellbeing.ti,ab OR exp well being OR exp mental health OR exp anxiety OR exp "depression (emotion)" OR exp posttraumatic stress disorder OR exp stress OR emotion*.ti,ab OR psycholog*ti,ab OR psychosocial.ti,ab OR socioemotional.ti,ab AND Exp parenting OR exp parents OR parent*.ti,ab OR mother*.ti,ab OR father*.ti,ab OR matern*.ti,ab OR patern*.ti,ab  “Ethnic groups”[mh] OR refugees[tiab] OR “displaced person*”[tiab] OR “emigrants and immigrants”[mh] OR migrant[mh] OR culture[mh] OR “culturally and linguistically diverse”[tiab] OR CALD[tiab] OR resettlement[tiab] AND “Perinatal care”[mh] OR “postnatal care”[mh] OR “prenatal education”[mh] OR “first 1000 days”[tiab] OR “first thousand days”[tiab] OR infant[mh] OR infant*[tiab] OR “child, preschool”[mh] OR “early life”[tiab] AND “Mental health”[mh] OR “stress, psychological”[mh] OR anxiety[tiab] OR wellbeing[tiab] OR “well being”[tiab] OR well-being[tiab] OR psychosocial[tiab] OR emotional[tiab] OR “stress disorders, traumatic”[mh] OR "posttraumatic stress disorder"[tiab] OR PTSD[tiab] OR “depression, postpartum”[mh] OR “postnatal depression”[tiab] AND Parenting[mh] OR parents[mh] OR parent*[tiab] OR mothers[mh] OR fathers[mh] OR matern*[tiab] OR pattern*[tiab]  Refugee* OR migrant* OR “culturally and linguistically diverse” OR CALD OR “asylum seeker*” OR immigrant* OR “displaced person*” OR resettlement AND Perinatal OR antenatal OR postnatal OR postpartum OR infant* OR “pre-school child*” OR “early life” OR pregnan* OR birth OR delivery OR “first 1000 days” OR “first thousand days” AND Wellbeing OR “well being” OR “mental health” OR anxiety OR depression OR “posttraumatic stress disorder” OR PTSD OR stress OR emotion* OR psycholog* OR psychosocial OR socioemotional OR "postnatal depression" AND Parenting OR parent* OR mother* OR father* OR matern* OR patern* |
