# Supplementary material for: Wellbeing Outcomes and Risk and Protective Factors for Parents with Migrant and Refugee Backgrounds from the Middle East in the First 1000 Days: A Systematic Review
Source: J Immigr Minor Health. 2023 Jul 6;26(2):395–408. doi: 10.1007/s10903-023-01510-4 (PMC10937786; doi:10.1007/s10903-023-01510-4)
Supplement: Supplementary file 2 — Supplementary Material 2 [file 10903_2023_1510_MOESM2_ESM.docx]

| Authors | Year | Aims | Study design | Sampling | Outcome focus/ measures/analysis | Main findings |
| --- | --- | --- | --- | --- | --- | --- |
| Ahmed, A.; Bowen, A.; Feng, C.X. | 2017 | To explore Syrian refugee women’s expectations of having a baby post-resettlement from a mental health perspective | Qualitatively-driven mixed methods: focus groups and survey | 12 Syrian refugee women living in Canada | - Maternal anxiety and depression (EPDS) - PTSD (Primary Care PTSD screening tool) - Intimate partner violence (WAST) - Sociodemographics - Descriptive analysis of survey and thematic analysis of focus groups | - Respondents reported understandings of maternal depression that differed from Western ideas - Social and spiritual support were important protective factors - Stigma and privacy concerns were barriers to help-seeking for respondents |
| Alhasanat, D.; Fry-McComish, J.; Yarandi, H.N. | 2017 | To determine the prevalence of and risk factors for PPD among Arabic migrant women | Quantitative cross-sectional survey via interview | 47 migrant and 3 refugee women from Yemen (*n*=24), Lebanon (*n*=15), Iraq (*n*=5), Egypt (*n*=1), Jordan (*n*=1), Qatar (*n=*1*)* and Saudi Arabia *(n*=1*)* living in USA | - Demographics - Maternal anxiety and depression (EPDS) - Risk factors using Postpartum Depression Predictors Inventory (PDPI-R) - Descriptive analysis and chi-square analysis of risk factors vs outcome | - Antenatal anxiety and/or depression, life stress, lack of social support, and maternity blues were significant risk factors for PPD |
| Alhasanat-Khalil, D.; Fry-McComish, J.; Dayton, C.; Benkert, R.; Yarandi, H.; Giurgescu, C. | 2018 | To examine relationships between acculturative stress, social support, and postpartum depression among Arabic migrant women | Quantitative cross-sectional survey | 111 migrant and 4 refugee women from Yemen (n=47), Lebanon (*n*=38), Iraq (*n*= 13) and other Arabic nations (*n*=17) living in USA | - Sociodemographics and medical history - Maternal anxiety and depression (EPDS) - Social support using Multidimensional Scale of Perceived Social Support (MSPSS) - Acculturative stress using Multi-Dimensional Acculturative Stress Inventory (MASI) - Data analysed using descriptive analysis, Pearson’s *r*, and stepwise regression | - Acculturative stress and lack of social support were predictors of PPD - High social support is related to lower rates of PPD - Social support did not moderate association between acculturative stress and PPD |
| Alhasanat-Khalil, D.; Giurgescu, C.; Benkert, R.; Fry-McComish, J.; Misra, D.P.; Yarandi, H. | 2019 | To examine the relationship between acculturation and postpartum depression among Arabic migrant women | Quantitative cross-sectional survey | 111 migrant and 4 refugee women from Yemen (n=47), Lebanon (*n*=38), Iraq (*n*= 13) and other Arabic nations (*n*=17) living in USA | - Sociodemographics and medical history - Maternal anxiety and depression (EPDS) - Acculturation using the Acculturation Rating Scale for Arabic Americans-II (ARSAA-II) - Data analysed using descriptive statistics, Pearson’s *r*, and multiple linear regression | - Acculturation levels did not predict PPD - Women who reported higher levels of marginalisation reported higher PPD symptoms |
| Alnuaimi, K.; Kassab, M.; Ali, R.; Mohammad, K.; Shattnawi, K. | 2017 | To compare pregnancy outcomes of Syrian refugee women and Jordanian women | Quantitative: retrospective cohort study | 644 Jordanian women; 616 Syrian refugee women living in north Jordan | - Sociodemographics - Birth data including mode of delivery, haemoglobin levels, pain relief, and maternal complications - Infant data collected but not extracted - Data analysed using chi-squared and independent samples t-tests | - Syrian mothers more likely to have caesarean delivery, foetal breech position, and be anaemic than Jordanian women. |
| Bawadi, H.; Ahmad, M.M. | 2017 | To explore the experience of childbirth and becoming a mother for Arab migrant women in the United Kingdom | Qualitative: hermeneutic phenomenology. Interviews in English or Arabic. | 8 migrant women from Jordan (*n*=2), Saudi Arabia (*n*=2), Algeria (*n*=1), Syria (*n*=1), Sudan (*n*=1) and Egypt (*n*=1), living in United Kingdom | - Discussion considered experiences and concerns during childbirth and motherhood - Cultural beliefs and practices - Hermeneutic phenomenological analysis through NVivo | - Participants reported emerging independence and individuality and a sense of self-determination - Emerging dominance of nuclear family over extended family |
| Bawadi, H., Al-Hamdan, Z. & Ahmad, M.M. | 2020 | To examine the challenges faced by Arab Muslim women in accessing maternity services and to suggest ways to improve the childbirth experience for them | Qualitative: hermeneutic phenomenology. Interviews in English or Arabic. | 24 interviews with 8 women from Jordan (*n*=2), Saudi Arabia (*n*=2), Algeria (*n*=1), Syria (*n*=1), Sudan (*n*=1) and Egypt (*n*=1), living in United Kingdom | - Discussion considered Discussion considered experiences and concerns during childbirth and motherhood - Hermeneutic phenomenological analysis through NVivo | - Participants expressed difficulties based on language and conflicting treatment practices - Participants reported instances of discrimination and prejudice from health care providers |
| Carlsson, T.; Balbas, B.; Mattson, E. | 2017 | To explore narratives of migrants with Arabic or Sorani interpreter needs when presented with a prenatal diagnosis of foetal anomaly | Qualitative: written accounts in Arabic. | 6 migrant women from Iraq (*n*=4), Jordan (*n*=1), Morocco (*n*=1), living in Sweden. | - Discussion considered experiences receiving diagnoses of foetal anomaly, and experiences with health care services - Responses translated into Swedish for content analysis | - Respondents experienced heightened emotions, particularly sorrow, loneliness, and shock - Respondents expressed fatalistic attitudes |
| Dadras, O., Nakayama, T., Kihara, M., Ono-Kihara, M., Seyedalinaghi, S. & Dadras, F. | 2021 | To explore the prevalence and associated sociodemographic factors of adverse pregnancy outcomes and examine the impact of intimate partner violence, food insecurity, poor mental health, and housing issues on pregnancy outcome in Afghan women living in Iran. | Quantitative cross-sectional survey | 424 Afghan women living in Iran. | - Sociodemographics - Intimate Partner Violence (yes/no) - Poor mental health (yes/no) - Housing issues (yes/no) - Food insecurity yes/no) - Antenatal care - Data analysed using descriptive analysis, bivariate analysis, and logistic regression | - Illiterate, unemployed, higher parity, lack of health insurance associated with higher adverse pregnancy outcomes. - Highest prevalence of obstetric complications: preterm labor, gestational hypertension, gestational diabetes, intrapartum haemorrhage. |
| di Ciano, T.; Rooney, R.; Wright, B.; Hay, D.; Robinson, L. | 2010 | To explore the postnatal support needs of Iraqi migrant women, in a group support setting | Qualitative: in-depth semi-structured interviews conducted in Arabic. | 7 women from Iraq, living in Australia | - Discussion considered participants’ understandings of their perinatal experiences, particularly through a cultural lens - Qualitative data analysed by interpretive phenomenological analysis. | - Respondents discussed the significance of motherhood, both in Iraq and Australia - Isolation and lack of social support, were reported as barriers to help-seeking, particularly for mental health concerns |
| Henry, J., Beruf, C. & Fischer, T. | 2020 | To investigate how premigration experiences, conceptions about pregnancy and birth, health literacy, and language skills influence access to healthcare, experiences of healthcare, and childbirth. | Qualitative: in-depth semi-structured interviews in Arabic (translated from German by interpreter) | 12 women from Syria (*n*=9), Iraq (*n*=2), and Palestine (*n*=1) | - Discussion considered concepts of pregnancy and childbirth, health literacy related to pregnancy, health promotion comparisons between Germany and participants’ home countries, and compensation mechanisms when access was impaired. - Content analysis applied | - Conceptions of pregnancy and childbirth differ among Arabic-speaking refugee women and influence health engagement behaviours and experiences of care. |
| Hjelm, K.; Bard, K.; Berntorp, K.; Apelqvist, J. | 2005 | To compare beliefs about health and illness between women born in Sweden and the Middle East who developed Gestational Diabetes | Qualitative: semi-structured interviews in Arabic. | 13 women born in Sweden and 14 migrant women from the Middle East living in Sweden; in their third trimester of pregnancy | - Discussion considered women’s knowledge of and beliefs regarding their diagnoses for Gestational Diabetes, and health behaviours - Qualitative data analysis not specified | - Women from the Middle East were more likely to focus on social and spiritual factors of GDM, to passively adapt to advice from professionals, and to express an external locus of control. - Swedish women were more likely to take an individualistic approach, to be active in management in hopes of it going away, and to express an internal locus of control |
| Hjelm, K.; Bard, K.; Berntorp, K.; Apelqvist, J. | 2009 | To explore beliefs about health and Gestational Diabetes; specifically whether they believe Gestational Diabetes is a prediabetic condition | Qualitative: semi-structured interviews in Arabic and Swedish | 13 women born in Sweden and 14 migrant women from the Middle East living in Sweden; three months post-partum | - Discussion considered women’s beliefs around health and illness, specifically causation and help-seeking - Analysis not specified | - Middle Eastern women indicated less knowledge and awareness of GDM, and expressed concerns around still being in a diabetic state. - Swedish women showed increased risk awareness, and were more likely to perceive GDM to be a risk marker for Type 2 Diabetes in the future. |
| Hjelm, K.; Bard, K.; Apelqvist, J | 2012 | To explore the development over time of beliefs about health, illness, and health care in women from the idle East and Sweden with a diagnosis of Gestational Diabetes | Qualitative: semi-structured interviews in Arabic and Swedish | 14 women from the Middle East (8 refugee; 6 migrant) living is Sweden. Includes data 12 months post-delivery | - Discussion considered women’s beliefs around health and illness related to gestational diabetes; specifically causation and help-seeking, self-care advice, and consequences of GDM - Analysis not specified | - Health behaviours of women changed over time - Participants required more information and follow-up care regarding gestational diabetes and diet than was required |
| Khawaja, M; Hammoury, N. | 2008 | To examine the correlates of forced sexual intercourse among pregnant refugee women attending an antenatal clinic in Sidon, Lebanon | Quantitative cross-sectional survey | 349 refugee women from Palestine living in Lebanon | - Sociodemographics - Abuse Assessment Screen - Sexual coercion (yes/no) - Results analysed using Pearson’s Chi-squared test and logistic regression | - 26.2% of women experienced sexual coercion in the past year - Sexual coercion significantly associated with lower education levels and fear of husband |
| Kiyak, H.; Gezer, S.; Ozdemir, C.; Gunkaya, S.; Karacan, T.; Gedikbasi, A. | 2020 | To compare delivery characteristics and short-term obstetric outcomes in Turkish women and Syrian refugee women | Quantitative: cross-sectional comparative study | 940 Turkish women; 616 Syrian refugee women | - Focus on birth outcomes: Caesarean delivery; APGAR scores; complications including pre-eclampsia, HELLP, placental abruption, pre- and post-term births - Independent samples t-test, chi-squared test, and Fischer’s exact tests used to compare variables. Logistic regression performed to identify risk factors. | - Turkish women more likely to deliver by caesarean, to deliver newborns <1000g or >4000g, and to experience pre-eclampsia, HELLP, of placental abruption. - No difference between groups for stillbirth or fetal anomaly rates, or APGAR scores. |
| Korukcu, O.; Aydin, R.; Conway, J.; Kukulu, K. | 2018 | To investigate the perinatal experiences of Syrian migrant women living in Turkey | Qualitative: in-depth semi-structured interviews in Turkish. | 7 Syrian refugee women living in Turkey. | - Discussion considered womens’ experiences during pregnancy and birth in Turkey - Results analysed by Thematic Analysis | - Respondents discussed fear relating to birth, which some women said was exacerbated by previous traumatic experiences - Women reported satisfaction with the maternity care they received in Turkey - Loneliness was reported as a result of being separated from family - Poverty was reported by respondents as exacerbating feelings of anxiety and discomfort |
| Miszkurka, M.; Goulet, L.; Zunzunegui, M.V. | 2010 | To compare the mental health of immigrant and Canadian women during pregnancy and assess the role of economics and social support in antenatal depressive symptomatology | Quantitative: cross-sectional cohort study | Sampling of Canadian (*n* = 3,834) and migrant women (*n* = 1,495). Middle Eastern women extracted (*n* = 70) | - Demographics - Center for Epidemiological Studies Depression Scale (CES-D) - Chi-squared tests of significance between regions of origin, length of stay, and CES-D scores | - 22% of Middle Eastern participants scored highly on CES-D, which was no different to Canadian women - Shorter length of time living in Canada associated with higher OR of depressive symptomatology in ME women than Canadian |
| Mohammad, K.I.; Awad, D.A.; Creedy, D.K.; Gamble, J. | 2018 | To investigate the prevalence of postpartum depression and associated factors among Syrian refugee women living in north Jordan | Quantitative cross-sectional | 365 Syrian refugee women living in north Jordan | - Sociodemographics - EPDS - Maternal Social Support Scale - Bivariate correlations and multiple regression | - Low social support, low monthly income, and recent arrival in Jordan were significantly associated with high EPDS scores |
| Nabolsi, M., Safadi, R., Sun, C., Ahmad, M., Al-Maharma, D., Halasa, S., Saleh, M. & Dohrn, J. | 2020 | To assess the HRQoL among Syrian refugee women of reproductive age living outside camps in Jordan. | Quantitative: correlational cross-sectional study. | 523 Syrian refugee women living in Jordan. | - Sociodemographics - HRQoL Short Form-36 (in Arabic) | - Significant negative correlations between HRQoL subdomains and sociodemographic factors - Strongest positive correlations between antenatal care and physical, role emotional, pain, and general health - Strongest negative correlations between length of marriage and pain, and between number of children and energy/fatigue. |
| Nahas, V.L.; Hillege, S. | 1999 | To explore the lived experiences of postpartum depression among Middle Eastern women living in Sydney | Qualitative: phenomenological study. In-depth unstructured interviews. | 45 women from the Middle East living in Australia | - Discussion considered women’s experiences of PPD, their understandings, and their help-seeking. - Analysed according to Colaizzi’s method of phenomenology | - Participants reported loneliness, lack of social support, and difficulty fulfilling gender role in Australia due to lack of support - Fear of being labelled a “bad” mother - Insufficient knowledge of PPD and available services |
| Ny P.; Plantin L.; Karlsson E.D.; Dykes A.-K. | 2007 | To describe Middle Eastern mothers’ experiences of maternal health services in Sweden, and the involvement of their male partner | Qualitative: Focus group discussions and individual semi-structured interviews | 13 women from Turkey, Syria, Iraq and Lebanon living in Sweden: 8 women in three focus groups conducted in Arabic; 5 individual interviews in Swedish (1 used Arabic interpreter). Some participants had refugee background (number not specified) | - Discussion considered women’s experiences of maternal health care and their partners’ involvement in maternity care. - Interviews analysed using content analysis | - Relationships with care providers varied but all women expressed the need for supportive, trustworthy providers and open communication. - Comfort levels at having partners present during discussions re: breastfeeding etc differed between women - Changes in family structure and division of labour post-resettlement were perceived in varied ways, but family support was considered important to all women |
| Ny, P.; Plantin, L.; Dejin-Karlsson, E.; Dykes, A.-K. | 2008 | To describe how men from the Middle East experience Swedish maternity and child health care | Qualitative: Focus group discussions and individual interviews | 16 men from the Middle East living in Sweden: 10 men in three focus groups conducted in Arabic; 6 individual interviews in Swedish | - Discussion considered men’s experiences with maternity care in Sweden, their involvement in care, and social constructions of the concept of fatherhood. - Content analysis | - Perceptions of provided information ranged from important, to “too much” and anxiety-inducing - Men felt respect for their partners as a result of being more involved - Dichotomy between new practices and role in family, and traditional role, practices, and beliefs - Being a role model was important, and sometimes a struggle when men found it difficult to find employment in Sweden |
| Peer M.; Soares CN.; Levitan RD.; Streiner DL.; Steiner M. | 2013 | To examine the factors associated with prenatal depressive symptoms, including altered HPA axis function, in Canadian immigrant women | Quantitative: cross-sectional survey and salivary cortisol assessment | Sampling of 78 migrant women living in Canada. Women from the Middle East extracted (*n* = 33) | - Sociodemographics - Psychiatric morbidty assessed with PRIME-MD PHQ - Perceived stress assessed using Perceived Stress Scale (PSS-10) - Perinatal depression screening using EPDS - Stressful life events assessed using Crisisin Family Systems-Revised (CRISYS-R) - Salivary cortisol samples collected - Analysis by Pearson’s *r* and MANOVA | - 24% of Middle Eastern participants reported high scores on EPDS - High EPDS scores across all countries of origin associated with higher perceived stress, more somatic symptoms, and lower social support - Salivary cortisol levels not reported across countries of origin |
| Qutranji, L., Silahli, N.Y., Baris, H.E. & Boran, P. | 2020 | To understand Syrian refugee women’s needs for care and the predisposing and enabling factors to healthcare access and utilisation. | Qualitative: in-depth semi-structured interviews | 47 Syrian refugee women living in Turkey. | - Discussion considered social life, mental health, language barriers, and accessing healthcare. - The Patient Health Questionnaire (PHQ-9) was administered to participants at the end of each interview. - Content analysis applied | - Participants reported social isolation, maternal depression, language barriers, and challenges in navigating the Turkish healthcare system. - Limited Turkish proficiency, limited education, and poor social resources exacerbated challenges. |
| Riggs, E.; Yelland, J.; Szwarc, J.; Wahidi, S.; Casey, S.; Chesters, D.; Fouladi, F.; Duell-Piening, P.; Giallo, R.; Brown, S. | 2016 | To explore the experiences of Afghan men of refugee background having a baby, and to report the reflections of service providers around the role of men in maternity settings | Qualitative: semi-structured interviews | 14 men and 16 women from Afghanistan with refugee backgrounds living in Australia; 34 health care providers working in Australia | - Discussion considered the fatherhood role and experiences with maternity and early childhood services - Providers discussed service needs of refugee families - Results analysed by Thematic Analysis | - Participants reported a change in role for men having babies in Australia, which can be difficult - A need to recognise and address men’s concerns in the health care setting was reported by respondents |
| Riggs, E.; Yelland, J.; Szwarc, J.; Duell-Piening, P.; Wahidi, S.; Fouladi, F.; Casey, S.; Chesters, D.; Brown, S. | 2020 | To explore the provision of health information for Afghan women and men during pregnancy, childbirth, and the first year after birth | Qualitative: semi-structured interviews | 14 men and 16 women from Afghanistan with refugee backgrounds living in Australia; 34 health care providers working in Australia | - Discussion considered participants’ experiences of maternity and early childhood health services - Results analysed by Thematic Analysis | - Respondents reported that a trusting relationship with health providers enables communication - Health providers reported employing varied methods of communication, such as diagrams/pictures - Respondents said family and friends are a common source of information for families - Cost, interpreter availability, fear of gossip, or concerns around sensitive topics reported by respondents as barriers to information |
| Russo A.; Lewis B.; Joyce A.; Crockett B.; Luchters S. | 2015 | To explore the experiences of Afghan women living in Melbourne throughout pregnancy, birth, and early motherhood | Qualitative: semi-structured individual interviews and focus groups | 38 Afghan women living in Australia: two focus groups (13 and 15 women), and 10 individual interviews | - Discussion considered transition to motherhood, giving birth, wellbeing and emotions, relationships, and differences between Afghanistan and Australia. - Results analysed using Thematic Analysis | - A high level of satisfaction with service provision was reported - Respondents reported tensions between traditional/familial advice and professional advice - Barriers to seeking support, particularly mental health support, were reported by respondents due to cultural reasons - Separation from traditional cultural beliefs and practices impacted respondents’ wellbeing - Emerging support role of men was reported - Social and community involvement reported as playing a key role in respondents’ emotional wellbeing |
| Shafiei, T.; Small, R.; McLachlan, H. | 2015 | To investigate immigrant Afghan women’s emotional well-being and experiences of postnatal depression after childbirth, and their use of health services | Mixed methods: in-depth semi-structured interviews and survey | 39 migrant women from Afghanistan living in Melbourne, Australia | - Sociodemographics - EPDS - Semi-structured interviews considered women’s emotional well-being and any experience of depression following birth - Descriptive statistics - Qualitative data analysed by Thematic Analysis | - 31% of women assessed as ‘probably depressed’ according to EPDS; 41% reported feeling depressed or very unhappy since having a baby - Respondents reported feeling overwhelmed, and reluctant to seek help. - Respondents had mixed experiences when they did seek professional help |
| Stevenson, K., Alameddine, R., Rukbi, K., Chahrouri, M., Usta, J., Saab, B., Bennett, P., Glover, V. & Reynolds, R.M. | 2019 | To estimate prevalence of depressive symptomatology among Syrian refugee mothers in Beirut, Lebanon | Quantitative: cross-sectional survey | 25 Lebanese women; 35 Syrian refugee women living in Lebanon | - Sociodemographics - EPDS - Domestic violence exposure (yes/no) - Results analysed using Pearson’s *r* and ANOVA | - While overall EPDS scores were high, 74.3% of Syrian mothers screened above 13 on EPDS, versus 52% of Lebanese mothers - Illegal residence, domestic violence, and previous mental illness were significant risk factors for higher EPDS scores |
| Stirling Cameron, E., Ramos, H., Aston, M., Kuri, M. & Jackson, L. | 2021 | To understand Syrian refugee women’s experiences accessing postnatal healthcare services and supports during the COVID-19 pandemic. | Qualitative: semi-structured interviews | 8 Syrian refugee women living in Canada | - Discussion regarding women’s experiences of having a baby during COVID-19 post-resettlement - Results analysed using elements of constructivist Grounded Theory | - Participants reported loss and grief surrounding expectations of pregnancy and birthing process due to COVID stay-at-home orders - Lost social supports and other forms of informal support exacerbated mental health concerns |
| Stirling Cameron, E., Aston, M., Ramos, H., Kuri, M. & Jackson, L. | 2022 | To understand Syrian refugee women’s perceptions and experiences of access to formal health services and informal supports during the postpartum period in Canada | Qualitative: semi-structured interviews | 11 Syrian refugee women living in Canada | - Discussion regarding women’s use of health services and experiences with health and social supports in Canada - Results analysed using elements of constructivist Grounded Theory | - Participants noted the importance of postpartum social support - Structural barriers and paternalistic health care providers impeded women’s access to services and decision-making autonomy |
| Urquia, M.L.; Ying, I.; Glazier, R.H.; Berger, H.; De Souza, L.R.; Ray, J.G. | 2012 | To evaluate women’s risk of preeclampsia across origin in migrant women in Canada | Quantitative: population-based cohort study | 118,849 migrant women. Women from the Middle East (including Maghreb region) extracted (*n* = 8552) | - Population-based data: women who migrated to Ontario 1985-2000 - Adjusted Odds Ratios and 95% CI calculated | - Women from the Middle East were not more likely to experience pre-eclampsia |
| Vigod, S.N.; Bagadia, A.J.; Hussain-Shamsy, N.; Fung, K.; Sultana, A.; Dennis, C.-L.E. | 2017 | To generate epidemiological evidence regarding differences in postpartum mental health care utilization of immigrant women in Ontario, Canada | Quantitative: population-based cohort study | 123,231 migrant women. Women from the Middle East (including Maghreb region) extracted (*n* = 15,182) | - Population-based data: migrant women who gave birth in Ontario 2008-2012 - Adjusted Odds Ratios and 95% CI calculated | - Women from the Middle East were more likely to have contact with mental health services than migrant women from North America or Europe - Women from Middle East were less likely to visit the emergency department or be hospitalized for mental health concerns than migrant women from North America or Europe |
| Yelland, J.; Riggs, E.; Wahidi, S.; Fouladi, F.; Casey, S.; Szwarc, J.; Duell-Piening, P.; Chesters, D.; Brown, S. | 2014 | To explore the responsiveness of health services to the social and mental health of Afghan women and men at the time of having a baby | Qualitative: semi-structured interviews | 14 men and 16 women from Afghanistan with refugee backgrounds; 34 health care providers | - Discussion considered issues around care provision including check-ups and tests, giving birth, interactions with care providers, length of hospital stay, home visiting by service providers, experience of maternal and child health services, and infant feeding. - Data analysed by Thematic Analysis | - Issues around gender of health care and interpreting providers - The majority of women reported being asked by health care providers about their circumstances; few were asked about relationship problems, violence at home, or financial or legal issues - Men were far less likely to report being asked about any issues related to social or emotional wellbeing. - Respondents said that identification of social health issues is important to providing care |
